# Molecular epidemiological characteristics of osteoarthritis-associated *Brucella melitensis* in China: evidence from whole-genome sequencing-based analysis

**DOI:** 10.1186/s12941-024-00671-w

**Published:** 2024-02-24

**Authors:** Lei Zhu, Chi Zhang, Chen Liang, Li Peng, Huanyu Yan, Xiuwen Liang, Youjia Xu

**Affiliations:** 1 The Second Affiliated Hospital of Soochow University, Soochow, China; 2 Hulunbuir People’s Hospital, Hulunbuir, China; 3 Hulunbuir City Sino-Mongolian Hospital, Hulunbuir, China

**Keywords:** Brucellosis, *Brucella melitensis*, Osteoarthritis, MLST, cgSNP analysis

## Abstract

**Background:**

Brucellosis, developing complications including arthritis, spondylitis, sacroiliitis, and osteomyelitis, is one of the most common zoonotic diseases in the current world which causes economic losses to the livestock industry and is a great public health concern. *Brucella melitensis* are the main pathogen of brucellosis epidemics in China, most of which are located in northern China. However, there is limited knowledge about the epidemiology of osteoarthritis-associated brucellosis. This study was aimed to reveal the prevalence of osteoarthritis-associated brucellosis in Inner Mongolia and also to investigate the molecular characteristics of *B. melitensis* isolates.

**Methods and results:**

In 2018, the osteoarthritis symptoms of brucellosis in the Brucellosis department of a hospital in Inner Mongolia were investigated. Twenty osteoarthritis-associated *B. melitensis* strains, isolated from the inpatients in Inner Mongolia during 2013–2017, were subjected to whole genome sequencing. The multilocus sequence type (MLST) and core genome SNP (cgSNP) analysis were conducted to detect molecular epidemiological characteristics. The incidence of brucellosis osteoarthritis symptoms in males (85/120, 70.8%) was significantly higher than that in females (35/120, 29.2%), and the age of patients was concentrated between 41 and 60 years old. In silico analyses indicated ST8 was the prevalent sequence type and the transmission of osteoarthritis-associated *B. melitensis* among different geographical areas. All strains carry virulence genes, including *cgs*, *lpsA*, *manCoAg*, *pgm*, *pmm*, *virB4*, *wbdA* and *wboA*.

**Conclusion:**

Our study showed the close epidemiologically connection of osteoarthritis-associated *B. melitensis* strains in northern China. And ST8 was the prevalent sequence type which need our attention.

**Supplementary Information:**

The online version contains supplementary material available at 10.1186/s12941-024-00671-w.

## Introduction

Brucella are non-motile, gram-negative, and facultative intracellular coccobacilli that could infect both humans and animals [1]. Brucellosis, a common zoonotic disease globally, is caused by *Brucella* spp. The genus *Brucella* comprises twelve highly genetically related species, and *B. melitensis* is the main pathogen of brucellosis epidemics in China [2]. The symptoms of Brucellosis are often non-specific, the most common symptoms of the acute form are fever, headache, backache, malaise, and anorexia [1]. It is often underreported, and misdiagnosed and once a chronic form develops, it could be resistant to treatment. Some cases could develop complications including arthritis, spondylitis, sacroiliitis, and osteomyelitis [3].

According to the reports, the Mediterranean region, the Middle East, and China shared the highest incidence of brucellosis [4]. In China, human brucellosis was first recorded in 1905 and was made statutorily notifiable in 1955 [5, 6]. In the late twentieth century, brucellosis was endemic in China, but it was effectively controlled by vaccination with attenuated *Brucella* vaccines [7]. However, brucellosis has re-emerged in China due to the ease of transporting animals [8–10]. At present, brucellosis is prevalent in 31 provinces or autonomous regions in China, most of which are located in northern China, where ruminant livestock is the primary source of income for people [11].

The epidemic status of brucellosis in Inner Mongolia has undergone drastic changes, and the incidence of human brucellosis has increased rapidly since 2010 [11]. During 2011–2016, Inner Mongolia was the region with the highest incidence rate of brucellosis in China, accounting for approximately 40% of reported cases [6, 12]. The epidemiology and incidence of brucellosis in this region represent the features of this disease in China, and *B. melitensis* has been the main species associated with human outbreaks [5, 13]. However, there is limited knowledge about the epidemiology of osteoarthritis-associated brucellosis in Inner Mongolia and the molecular genetic characteristics of *B. melitensis* that induce osteoarthritis is still unclear. This study aimed to reveal the prevalence of osteoarthritis-associated brucellosis in Inner Mongolia and also to investigate the molecular characteristics of osteoarthritis-associated *B. melitensis* isolates.

## Materials and methods

### Epidemiological data collection

This study including inpatients in the Brucellosis department of a hospital in Inner Mongolia in 2018. Patients with osteoarthritis symptoms of brucellosis were selected for data analysis. Data on brucellosis cases were collected from the online National Notifiable Infectious Disease Reporting Information System of the Chinese Center for Disease Control and Prevention.

### Strain isolation and identification

A total of twenty osteoarthritis-associated *B. melitensis* strains from the inpatients in Inner Mongolia were included in this study which were isolated during 2013–2017. Strains were isolated from the blood or joint fluid samples. The species identification was conducted under the procedures recommended by WS 269-2019 guidelines of China.

### Whole genome sequencing (WGS) and in silico analyses

All twenty *B. melitensis* strains were subjected to whole genome sequencing. Briefly, total DNA was extracted using the Gentra Puregene Yeast/Bact. Kit (Qiagen, Dusseldorf, Germany) and sequenced on the Illumina NovaSeq 6000 (San Diego, CA, USA) platform. The assembly of the complete genome was performed using SPAdes v3.10.1 [14]. The average nucleotide identity (ANI) analysis of twenty strains was conducted with pyani (https://github.com/widdowquinn/pyani). The multilocus sequence types were identified by the software mlst (https://github.com/tseemann/mlst). Additionally, the virulence genes and acquired antimicrobial resistance genes (ARGs) of strains was detected using VFDB and ResFinder (https://cge.cbs.dtu.dk/services/ResFinder/), respectively [15].

### Core genome SNP (cgSNP) analysis

Core genome single-nucleotide polymorphism (cgSNP) analysis was conducted with Snippy v4.6.0, with the *B. melitensis* 16 M (GCA_000007125.1) as the reference. Recombination events, mobile genetic elements (MGEs) and putative repetitive sections were filtered using Gubbins v.2.4.1. The maximum likelihood tree was constructed by FastTree v2.1.10 and visualized by iTOL [16]. The SNP distance between each pair of strains was calculated by snp-dists v0.4.

To evaluate the relatedness of ST8 *B. melitensis* global, sixty ST8 *B. melitensis* genomes were downloaded from NCBI database. And the cgSNP analysis was performed with these genomes (genomes from this study and NCBI database). The *B. melitensis* 16 M (GCA_000007125.1) used as the reference.

## Results and discussion

In 2018, a total of 235 patients hospitalized for brucellosis, of whom 120 had brucellosis osteoarthritis symptoms. The positive rate was 51.1%. As shown in Table 1, the incidence in males (85/120, 70.8%) was significantly higher than that in females (35/120, 29.2%). In addition, the age of patients was concentrated between 41 and 60 years old (74/120, 61.7%) which was consistent with a previous study in Guizhou Province, China [17].

**Table 1:** The numbers and percentages (%) of 120 osteoarthritis-associated *B. melitensis* isolates

	Gender		Age			
	Male	Female	≤ 20	21–40	41–60	> 60
No.	85	35	2	31	74	13
%	70.8	29.2	1.7	25.8	61.7	10.8

**Fig. 1:**
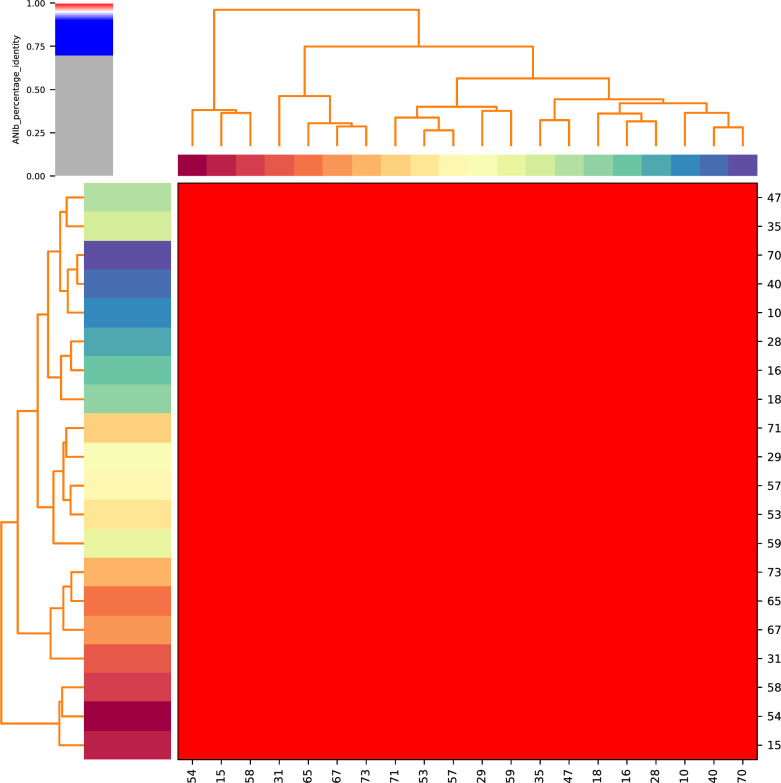
The average nucleotide identity profiles of twenty *B. melitensis* isolates in this study

**Fig. 2:**
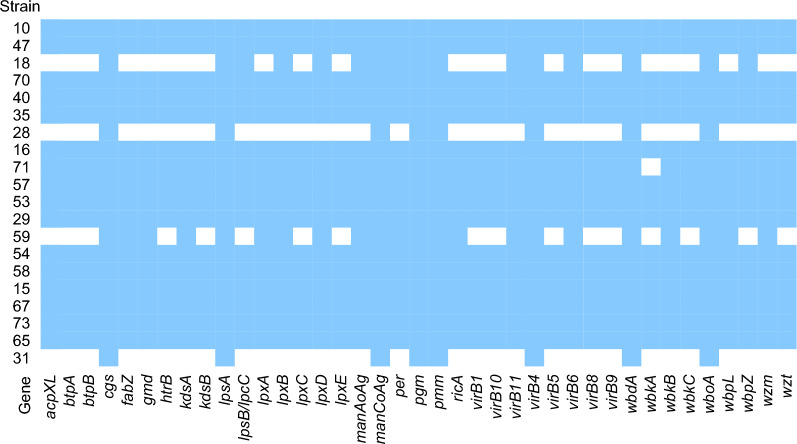
The heat map of virulence genes in this study

To explore the molecular epidemiological characteristics of osteoarthritis-associated *B. melitensis* further, we sequenced twenty *B. melitensis* isolates, which were isolated from 2013 to 2017. All genomes were uploaded to NCBI under the BioProject: PRJNA1015399. Unlike other genus, *Brucella* is extremely homologous, with identity greater than 90% [18]. According to the results of ANI analysis, twenty isolates in this study shared the identity over 95% (Fig. 1). The highly conserved genome poses a great challenge for the typing of genus *Brucella*. Multilocus sequence typing (MLST) is a trustworthy method to characterizing for *Brucella* spp. populations [19]. In this work, all strains, based on 21 loci MLST technology, were ST8. ST8 is a common type mainly distributed in Asia, Europe, and Africa [17]. In previous study, ST8 *B. melitensis* causing a brucellosis epidemic in Qinghai, China [20]. VFDB database was used to analyze the virulence genes carried by the twenty strains. The results showed that all the twenty strains carried a variety of virulence genes, and genes *cgs*, *lpsA*, *manCoAg*, *pgm*, *pmm*, *virB4*, *wbdA* and *wboA* were carried by all strains (Fig. 2). According to the results of ResFinder, no strain carries ARGs.

According to previous report, WGS-based analysis has been shown to distinguish closely related *B. melitensis* strains and can discriminate intraspecies relationships [21].

**Fig. 3:**
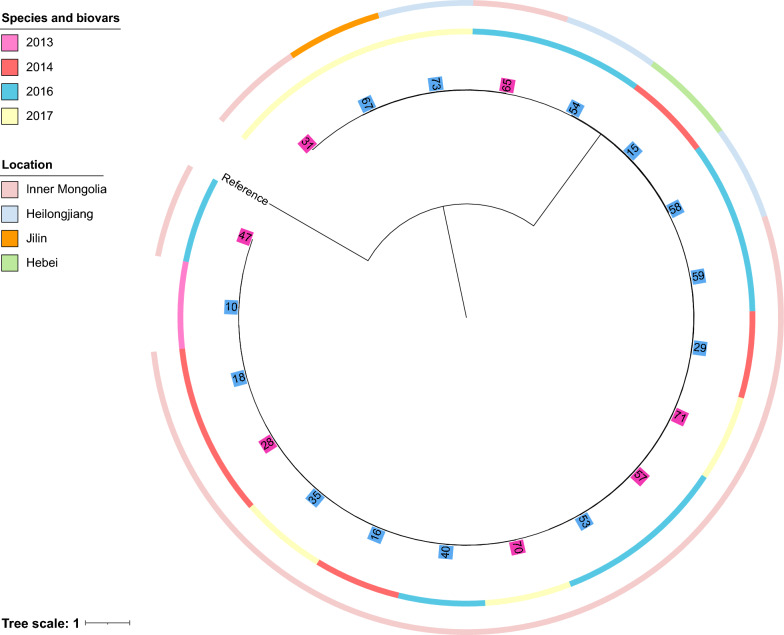
Maximum likelihood tree based on cgSNP alignment of osteoarthritis-associated *B. melitensis* strains. *B. melitensis* 16 M (GCA_000007125.1) was used as the reference. The year and location of isolation are also given. The gender of the host was labeled by different color, pink represent female and blue represent male

**Fig. 4:**
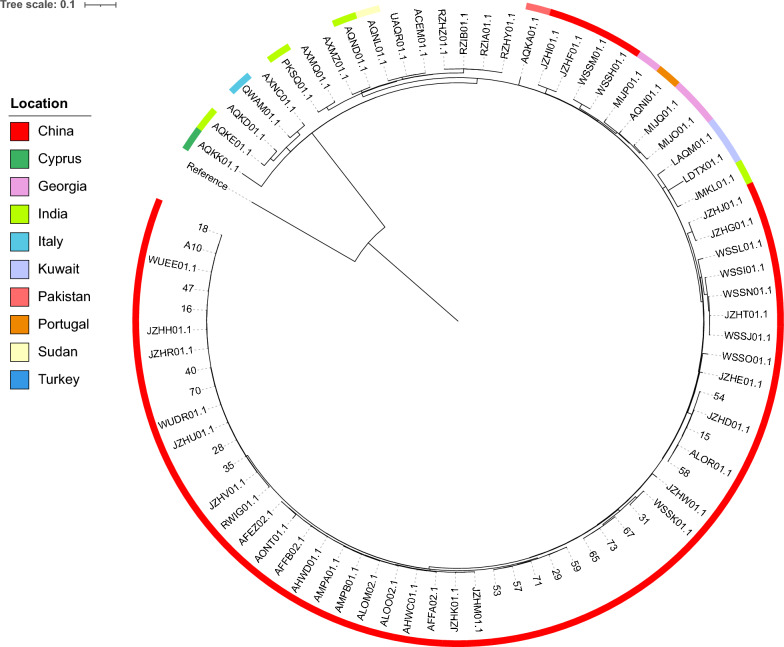
Maximum likelihood tree based on cgSNP alignment of ST8 *B. melitensis* strains. *B. melitensis* 16 M (GCA_000007125.1) was used as the reference. The location of isolation was given

Based on the Fig. 3 and Additional file 1: Table S1, *B. melitensis* in this study shared limited SNPs difference. Among them, the isolates 53 and 57, which were both isolated from Inner Mongolia in 2016, showed the highest identity (one SNP difference). What’s more, seven pairs of genomes in this study differed less than ten SNPs. *B. melitensis* 65 that was isolated from Inner Mongolia in 2016 differed in six SNPs from strains 73 and 67, which were isolated in Heilongjiang and Jilin in 2017, respectively. Globally, Inner Mongolia strains in this study shared a high identity, suggesting they may have developed from a common ancestor. Moreover, to evaluate the relatedness of ST8 *B. melitensis* global, a ML tree was constructed based global genomes (Fig. 4). China was the most common country isolated ST8 *B. melitensis.* In addition, most of the strains isolated from China clustered together. Isolates from different geographical regions exhibit similar epidemiological features, indicating the transmission among different regions and requiring our attention to strengthen prevention and control.

## Conclusion

To gain a further understanding of the epidemiology of osteoarthritis-associated Brucellosis, the molecular characteristics of *B. melitensis* strains in northern China (Inner Mongolia, Heilongjiang and Jilin) were examined. Our study showed the close epidemiologically connection of osteoarthritis-associated *B. melitensis* strains based on cgSNP analysis. ST8 was the prevalent sequence type which need our attention. All in all, we need to take more effective measures to prevent and control brucellosis in the future.

### Supplementary Information

**Additional file 1: Table S1.** Pairwise cgSNPs in this study.

## Data Availability

All genomes in this study were uploaded to NCBI under the BioProject: PRJNA1015399.

## Abbreviations

*B. melitensis*: *Brucella melitensis*
ANI: Average nucleotide identity
MLST: Multilocus sequence typing
cgSNP: Core genome single-nucleotide polymorphism
MGEs: Mobile genetic elements
